# Use of Electrocardiography to Predict Future Development of Hypertension in the General Population

**DOI:** 10.1097/MD.0000000000003483

**Published:** 2016-04-29

**Authors:** Hiroyuki Takase, Tomonori Sugiura, Shunsuke Murai, Sumiyo Yamashita, Nobuyuki Ohte, Yasuaki Dohi

**Affiliations:** From the Department of Internal Medicine (HT), Enshu Hospital, Hamamatsu; Department of Cardio-Renal Medicine and Hypertension (TS, SM, SY, NO), Nagoya City University Graduate School of Medical Sciences, Nagoya; and Department of Internal Medicine (YD), Faculty of Rehabilitation, Nagoya Gakuin University, Seto, Japan.

## Abstract

Cardiac muscle responds to increased afterload by developing hypertrophy. During the early stages of hypertension, the heart can be transiently, but frequently, exposed to increased afterload. This study was designed to test the hypothesis that left ventricular hypertrophy (LVH) assessed by electrocardiography (ECG) can be used to predict future development of hypertension.

Sokolow–Lyon voltage and Cornell product were calculated using ECG in 5770 normotensive participants who visited our hospital for a physical checkup (age 52.7 ± 11.3 years). LVH was defined as a Sokolow–Lyon voltage of >3.8 mV or a Cornell product of >2440 mm × ms. After baseline examination, participants were followed up with the endpoint being the development of hypertension.

During the median follow-up period of 1089 days (15,789 person-years), hypertension developed in 1029 participants (65.2/1000 person-years). A Kaplan–Meier analysis demonstrated a significantly higher incidence of hypertension in participants with LVH than in those without LVH as assessed by Sokolow–Lyon voltage or Cornell product (*P* < 0.0001 for both). The hazard ratios for incident hypertension in participants with LVH defined by Sokolow–Lyon voltage and Cornell product were 1.49 (95% confidence interval [CI] 1.16–1.90, *P* < 0.01) and 1.34 (95% CI 1.09–1.65, *P* < 0.01), respectively, after adjustment for possible risk factors. Furthermore, in multivariable Cox hazard analysis, where Sokolow–Lyon voltage and Cornell product were taken as continuous variables, both indices were independent predictors of future hypertension (*P* < 0.0001).

Both Sokolow–Lyon voltage and Cornell product are novel predictors of future development of hypertension in the general population.

## INTRODUCTION

Hypertension is a major risk factor for cardiovascular events.^1–3^ Although the increased risk of hypertension in individuals can be reduced, at least to some extent, by reducing blood pressure,^4–6^ only ∼50% of hypertensive patients are under medical treatment, and more than half of all cardiovascular events occur in individuals with high normal blood pressure or mild hypertension.^3,7–9^ Thus, primary prevention of hypertension is an attractive approach to reducing cardiovascular morbidity and mortality. A recent clinical trial demonstrated that individuals with prehypertension, who were treated with an angiotensin receptor blocker for 2 years, had a 15% reduction in the incidence of hypertension over 4 years compared with those administered a placebo.^10^ Furthermore, modification of lifestyle,^11,12^ a low-salt diet,^12–14^ and regular physical exercise^12^ reduce the incidence of hypertension. Although the feasibility and efficacy of preventing hypertension has been demonstrated, a strategy that targets all individuals without hypertension would promote sustainable use of medical and economic resources. An individualized approach of risk stratification and targeted treatment of the normotensive individuals who are at greatest risk of progression to hypertension might therefore be more effective in preventing the development of hypertension among the general population.

The heart is a key target organ of high blood pressure, and cardiac muscle responds to increased afterload (i.e., systemic blood pressure) by developing hypertrophy. Blood pressure gradually increases, with significant fluctuations, during the development of hypertension, indicating that the heart may be transiently, but frequently, exposed to increased afterload during the earlier stages of hypertension. Thus, even a mild increase in electrocardiogram voltage that indicates an increase in left ventricular mass could be an early symptom of developing hypertension. In this study, we tested the hypothesis that left ventricular hypertrophy (LVH) assessed by electrocardiography (ECG) can be used to predict future development of hypertension in the normotensive general population.

## METHODS

### Study Design

A prospective cohort study was conducted using apparently healthy persons who participated in our yearly physical checkup program from July 2008 to June 2013 to investigate the impact of LVH assessed by ECG on the incidence of hypertension. The study was performed in accordance with the principles of the Declaration of Helsinki, and the study protocol was approved by the ethics committee of the Enshu Hospital (Hamamatsu, Japan). All the subjects gave written informed consent to participate prior to the start of the study and at each study visit.

### Study Participants and Procedures

The study participants were recruited from the group of participants who visited our hospital for a yearly physical checkup between July 2008 and December 2011 (n = 9018). First, participants under medical treatment for hypertension (n = 2032) were excluded. The checkup program, then, indicated that 6095 participants among the remaining 6986 participants did not have hypertension.

Participants with wide QRS (≥0.12 secconds), such as left or right bundle branch block and Wolff–Parkinson–White syndrome, were also excluded. Other exclusion criteria for the study were myocardial infarction, cardiac myopathy, heart failure, and disorders requiring medication that may affect blood pressure. The remaining 5944 participants were enrolled and followed up with the endpoint being the onset of hypertension. Because data beyond the baseline measurements were not available for 174 participants, data from the remaining 5770 participants were analyzed (follow-up rate 97.1%). LVH was defined as a Sokolow–Lyon voltage of >3.8 mV^15,17^ and Cornell product of >2440 mm × ms.^16–19^

The yearly increase in blood pressure was obtained in each participant from the linear regression analysis where a change in blood pressure and the follow-up period (in years) was taken as the dependent and independent variable, respectively. The slope of the regression line was considered to be the yearly increase in blood pressure. The regression analysis was performed using data obtained before the start of antihypertensive drugs in participants who developed hypertension during the study period. The impact of baseline LVH, defined by Sokolow–Lyon voltage and Cornell product, on the onset of hypertension was investigated. As part of the annual health checkup program, blood pressure was measured using a mercury sphygmomanometer after participants had been seated in a chair for 5 minutes with their backs supported. Blood pressure was measured for 3 times at an interval of 2 minutes, and the mean of the second and third measurements was taken as the blood pressure. Participants were classified as having hypertension if their systolic blood pressure was ≥140 mmHg, if their diastolic blood pressure was ≥90 mmHg, or if they used antihypertensive medications.^3^ Participants were defined as having dyslipidemia if their high-density lipoprotein cholesterol level was <40 mg/dL, if their low-density lipoprotein (LDL) cholesterol level was ≥140 mg/dL, if their triglyceride level was ≥150 mg/dL, or if they used antidyslipidemic medications.^20^ Participants were defined as having diabetes mellitus if their fasting plasma glucose level was ≥126 mg/dL or if they used antidiabetic medications. The estimated glomerular filtration rate (eGFR) was calculated using the Chronic Kidney Disease Epidemiology Collaboration equation.^21^

### Statistical Analysis

The software package IBM SPSS statistics 18 (Chicago, IL) was used for all analyses. All data except for B-type natriuretic peptide (BNP) and the follow-up period are expressed as the mean ± standard deviation. BNP and the follow-up period are expressed as the median with interquartile range. Because the distribution of BNP was skewed rightward, BNP was log-transformed before statistical analysis. Differences between 2 means that had a normal distribution were compared using the unpaired Student *t* test. The significance of difference in follow-up periods was assessed by the Mann–Whitney *U* test. Comparisons between categorical data were assessed by the Yates corrected χ^2^ test. The Kaplan–Meier method was used for calculating cumulative incidence rates of new-onset hypertension. To analyze the endpoint throughout the observation period, the significance of differences in the cumulative incidence rates was evaluated by the log-rank test and adjusted by multivariate Cox proportional hazard regression models. Hazard ratios and 95% confidence intervals (CIs) were calculated. Log–log plots confirmed the proportional hazards assumption. Furthermore, the relationship of Sokolow–Lyon voltage and Cornell product as continuous variables with the onset of hypertension was examined using multivariate Cox proportional hazard regression models. In other series of analyses, the relationships between baseline Sokolow–Lyon voltage and changes in blood pressure and between baseline Cornell product and changes in blood pressure were investigated by multivariate linear regression analysis. *P* < 0.05 was considered significant.

## RESULTS

The baseline characteristics of all participants are shown in Table 1. Cross-sectional analysis at baseline indicated that Sokolow–Lyon voltage and Cornell product showed significant correlations with many other variables including blood pressure in univariate models (Table 2). Both indices obtained from ECG remained significant predictors of systolic blood pressure (*r* = 0.172 and *r* = 0.166, respectively; both *P* < 0.001) after adjustment for age, sex, body mass index, heart rate, eGFR, uric acid, fasting plasma glucose, LDL cholesterol, triglyceride, BNP, and hemoglobin.

**TABLE 1:**
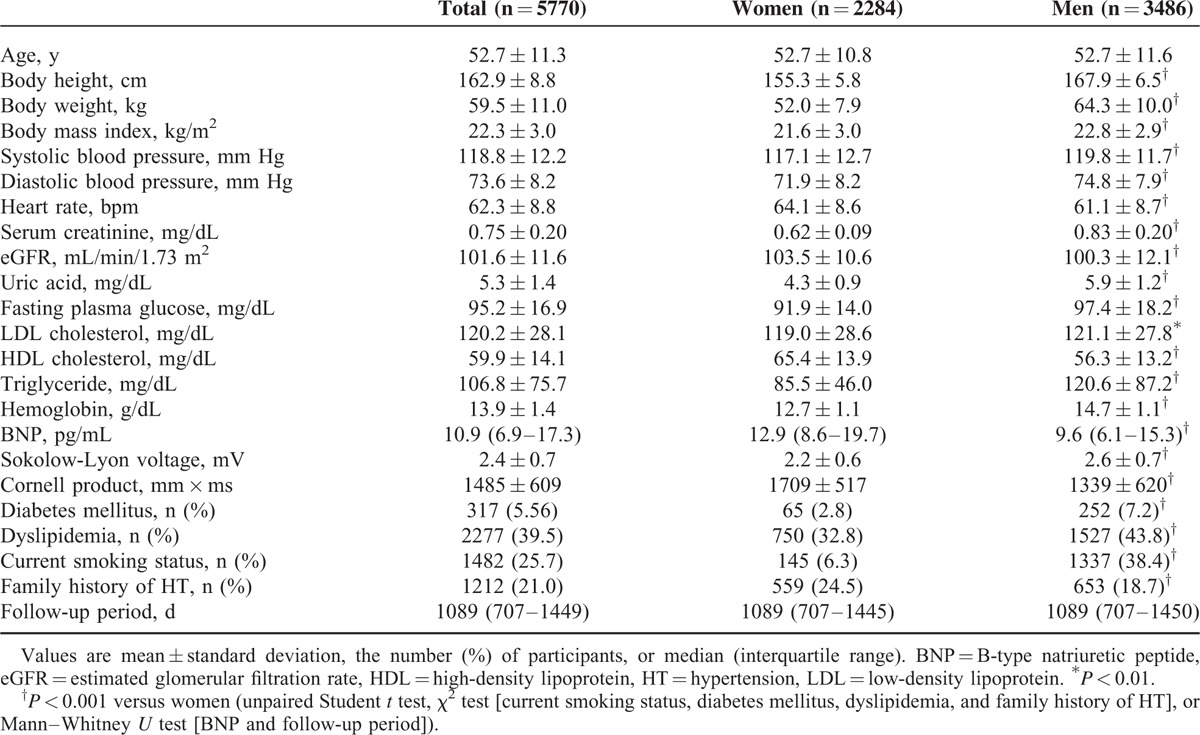
Baseline Characteristics of the Study Subjects

**TABLE 2:**
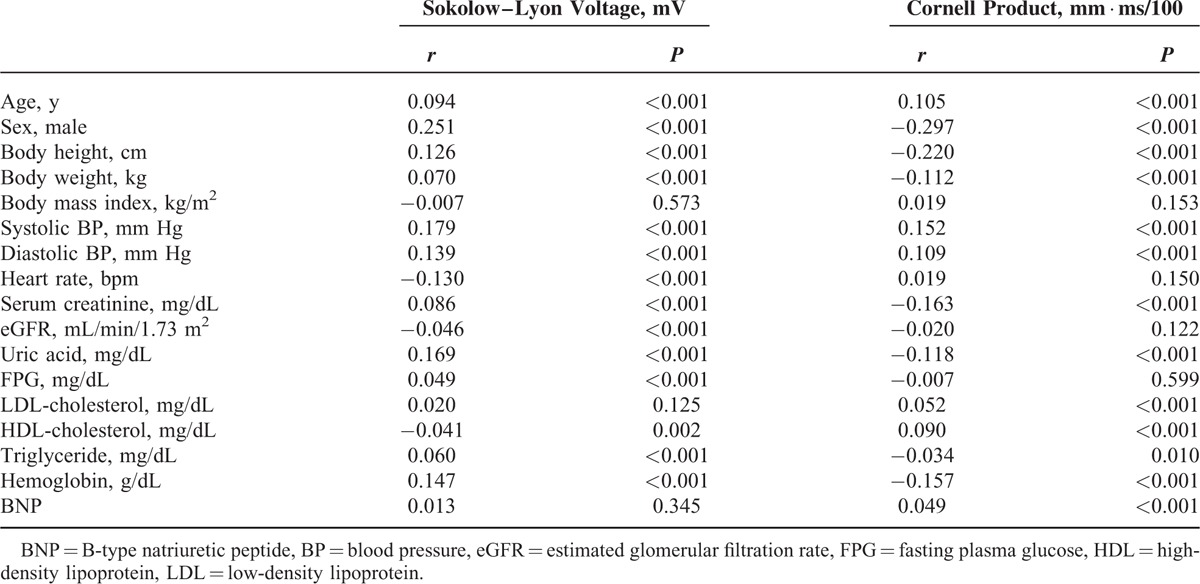
Relationship Between Sokolow–Lyon Voltage or Cornell Product and Other Variables at Baseline (Univariate Analysis)

The follow-up period of was 15,789 person-years, and the median follow-up period per participant was 1089 days (range 168–1818 days). During the follow-up period, hypertension developed in 1029 participants (17.8%, 65.2/1000 person-years) with a higher incidence in men (21.3%, 77.4/1000 person-years) than in women (12.6%, 46.3/1000 person-years). Table 3 describes the results of retrospective analysis, showing characteristics of participants with and without future development of hypertension. The mean Sokolow–Lyon voltage and Cornell product values at baseline were higher for participants who developed hypertension than for participants who did not develop hypertension (Table 3).

**TABLE 3:**
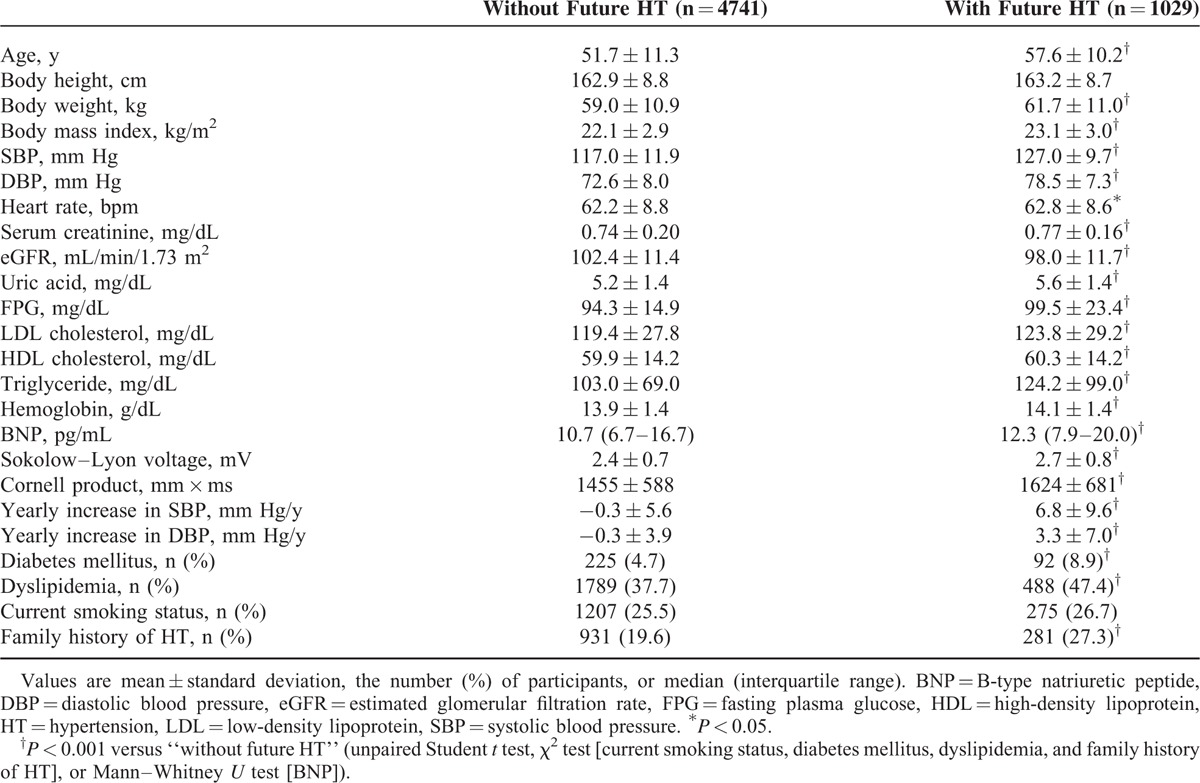
Retrospective Analysis of Study Subjects’ Characteristics at Baseline

**FIGURE 1:**
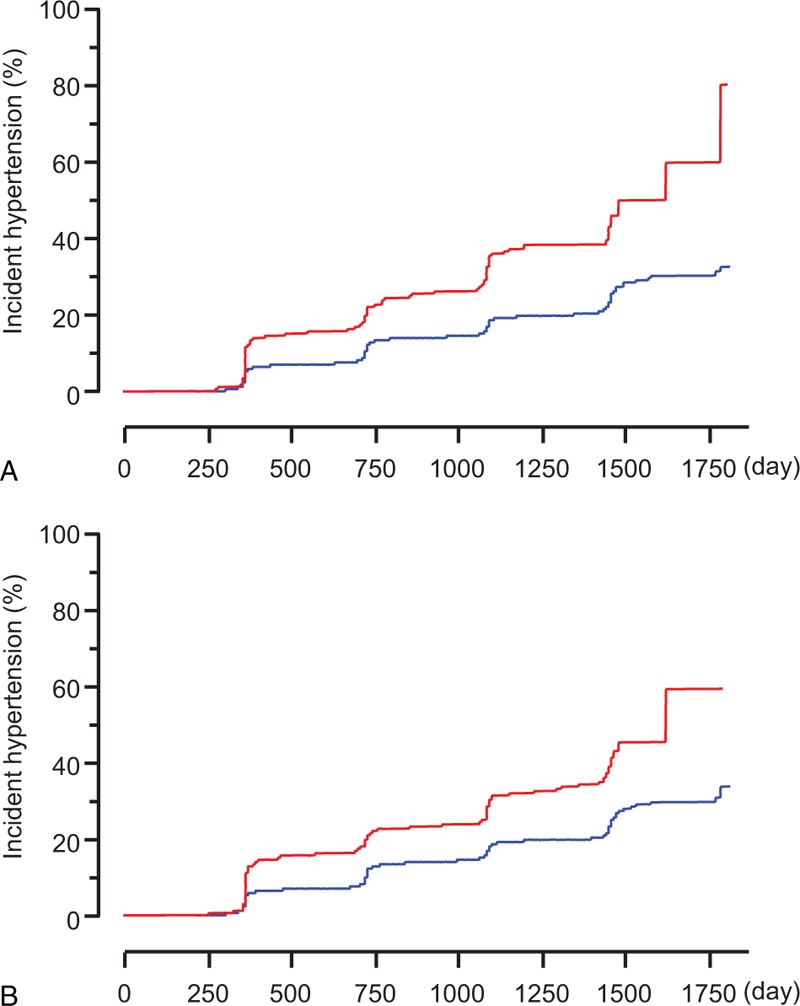
Plots of incidence rates of hypertension in participants with (red lines) and without (blue lines) LVH at baseline defined by (A) Sokolow–Lyon voltage (>3.8 mV, n = 211) and (B) Cornell product (>2440 mm × ms, n = 366). *P* < 0.0001 by log-rank test. LVH = left ventricular hypertrophy.

**FIGURE 2:**
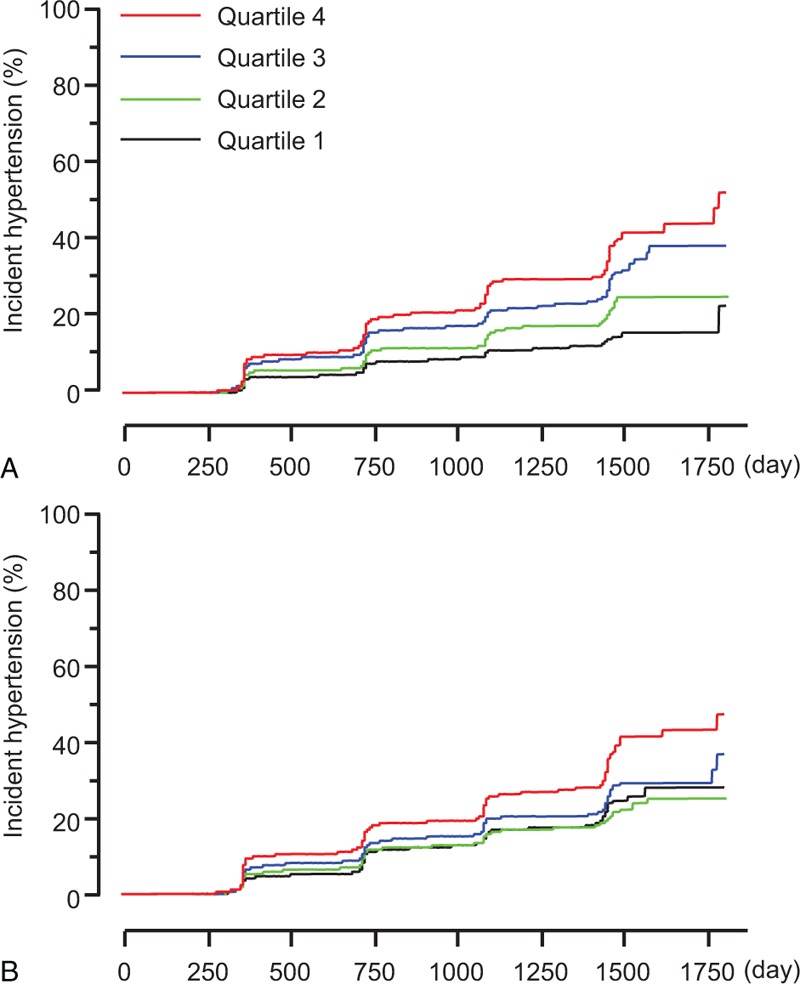
Plots of incidence rates of hypertension. Participants were divided into the quartiles according to their Sokolow–Lyon voltage (A) or Cornell product (b) levels at baseline. *P* < 0.0001 by log-rank test.

To evaluate the impact of LVH (assessed using Sokolow–Lyon voltage or Cornell product) on the incidence of hypertension, participants were divided into 2 groups: those with and without LVH at baseline. Analysis of the plot of cumulative incidence rate of hypertension demonstrated that the incidence of hypertension was significantly higher in participants with LVH at baseline than in those without LVH at baseline using both Sokolow–Lyon voltage (133.3 vs 62.6/1000 person-years) and Cornell product (116.4 vs 61.8/1000 person-years) (Figure 1). The hazard ratios for incident hypertension for LVH defined by Sokolow–Lyon voltage and Cornell product were 1.49 (95% CI 1.16–1.90) and 1.34 (95% CI 1.09–1.65), respectively, after adjustment for age, sex, body mass index, systolic blood pressure, heart rate, eGFR, uric acid level, fasting plasma glucose level, LDL cholesterol level, triglyceride level, BNP level, smoking status, and family history of hypertension. The incidence of hypertension was also increased across the quartiles of Sokolow–Lyon voltage (35.2, 52.4, 74.7, and 98.7/1000 person-years in the first, second, third, and fourth quartiles, respectively) and those of Cornell product (54.6, 52.0, 65.3, and 89.5/1000 person-years in the first, second, third, and fourth quartiles, respectively) (Figure 2). The hazard ratio of incident hypertension (first quartile as reference) in the second, third, and fourth quartiles was 1.31 (95% CI 1.04–1.64), 1.59 (95% CI 1.29–1.98), and 1.76 (95% CI 1.42–2.18), respectively, for Sokolow–Lyon voltage, and 1.06 (95% CI 0.87–1.29), 1.26 (95% CI 1.04–1.52), and 1.49 (95% CI 1.23–1.79), respectively, for Cornell product after adjustment for age, sex, body mass index, systolic blood pressure, heart rate, eGFR, uric acid, fasting plasma glucose, LDL cholesterol, triglyceride, BNP, current smoking habit, and family history of hypertension. Then, Sokolow–Lyon voltage and Cornell product values at baseline were taken as continuous variables. A correlation between Sokolow–Lyon voltage at baseline and the future incidence of hypertension and between Cornell product at baseline and the future incidence of hypertension was evident in univariate Cox hazard regression analysis (Table 4). Furthermore, multivariate Cox hazard analysis revealed that Sokolow–Lyon voltage and Cornell product were independent predictors of incident hypertension (Table 5).

**TABLE 4:**
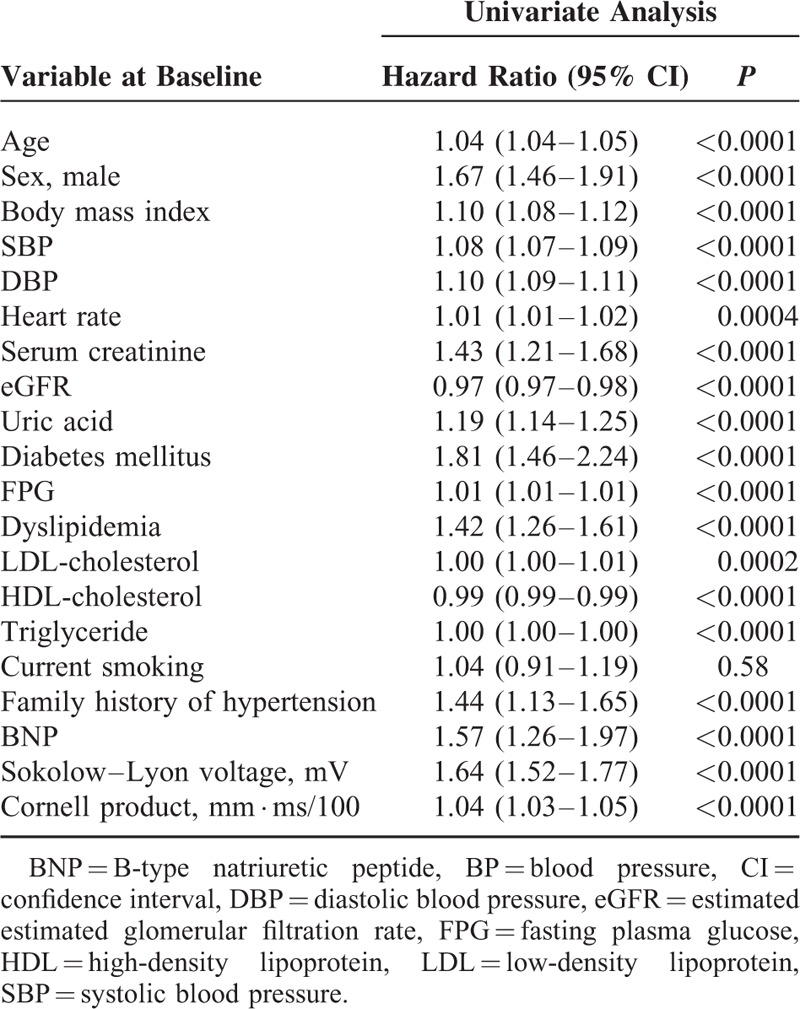
Univariate Cox Proportional Hazard Regression Analyses for Future Development of Hypertension

**TABLE 5:**
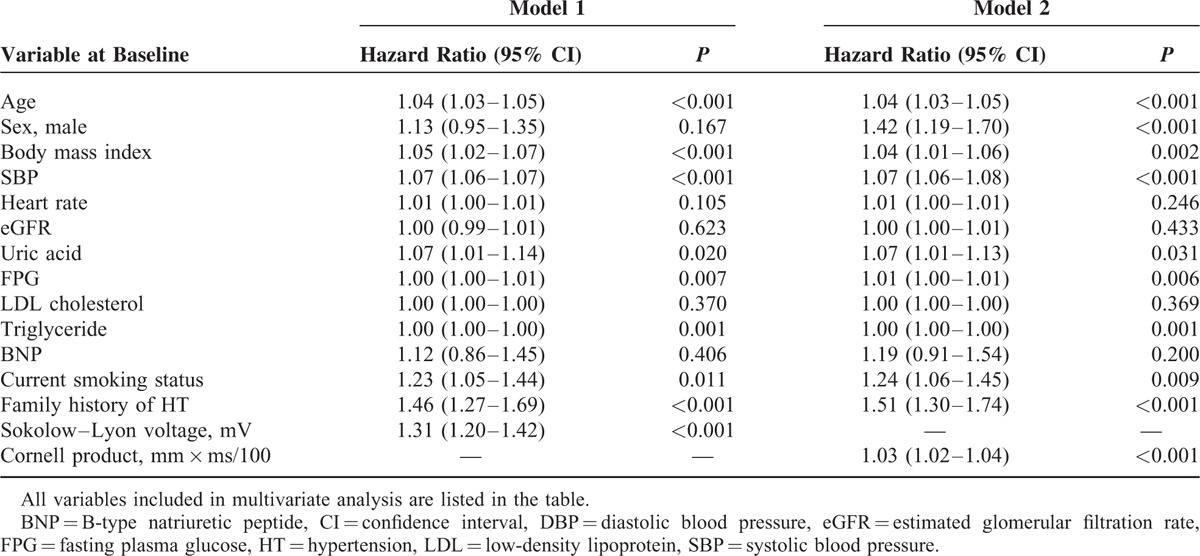
Multivariate Cox Proportional Hazard Regression Analyses for Future Development of Hypertension

The impact of Sokolow–Lyon voltage and Cornell product at baseline on changes in systolic blood pressure was also investigated. Both Sokolow–Lyon voltage and Cornell product positively correlated with a yearly increase in systolic blood pressure (Table 6). Similar results were obtained when a yearly increase in diastolic blood pressure was used as a dependent variable (data not shown).

**TABLE 6:**
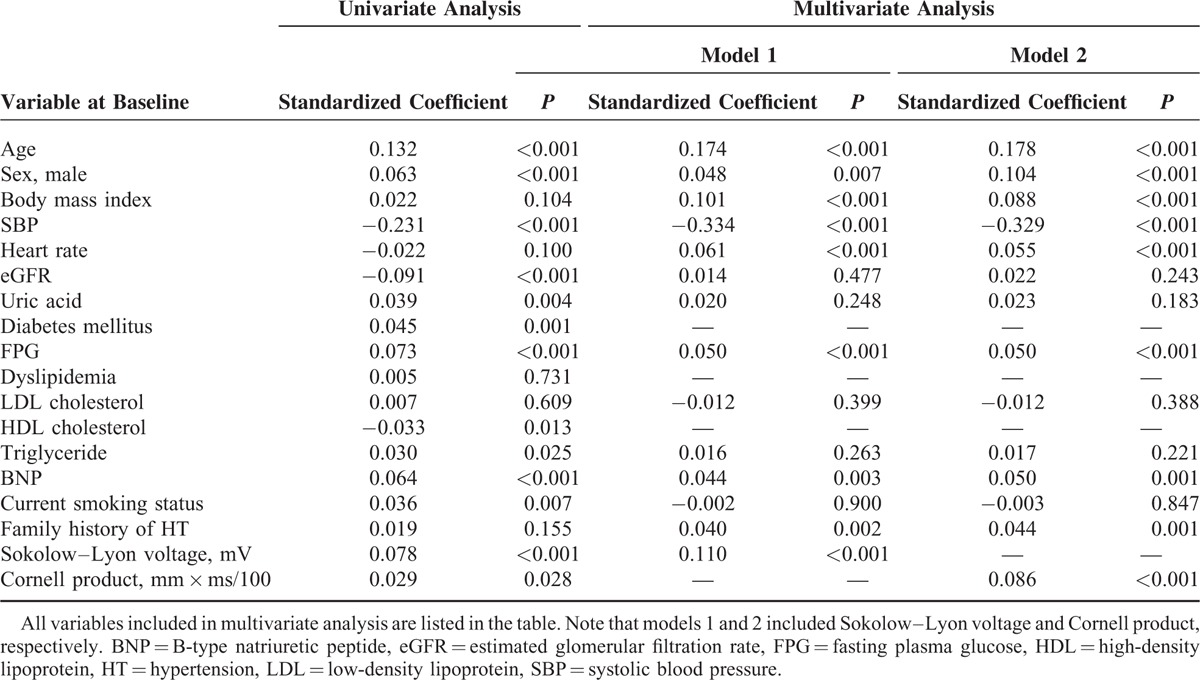
Univariate and Multivariate Regression Analyses Demonstrating the Relationship Between Baseline Variables and Yearly Increase in Systolic Blood Pressure

Indices obtained from ECG were not dramatically changed both in participants who developed (Sokolow–Lyon voltage −0.06 ± 0.35 mV/year, Cornell product 14 ± 237 mm × ms/year) and did not develop hypertension during the study period (−0.08 ± 0.34 mV/year and 4 ± 182 mm × ms/year, respectively), and the yearly change was not different between these 2 groups.

## DISCUSSION

We found herein that the presence of LVH assessed by Sokolow–Lyon voltage or Cornell product predicts the future development of hypertension in the general Japanese population of individuals without hypertension. These results strongly suggest that these indices obtained by ECG, which are closely related with future increases in blood pressure and the incidence of hypertension, are novel markers of increased cardiovascular risk.

The analysis of cumulative incidence rates of hypertension showed a significantly higher incidence of hypertension in individuals with LVH than in those without LVH, defined by Sokolow–Lyon voltage or Cornell product criteria. Indeed, the risk of developing hypertension was increased by ∼50% in individuals with LVH after adjustment for important factors including baseline blood pressure, indicating that LVH defined by ECG is a powerful predictor of hypertension onset. The concept that hypertension is predictable using ECG was confirmed by the finding of increased risk of developing hypertension across the quartiles of Sokolow–Lyon voltage and Cornell product. In line with our hypothesis, Sokolow–Lyon voltage and Cornell product values used as continuous variables were closely associated with the incidence of hypertension and correlated with increases in blood pressure during the follow-up period independently of several important factors measured at baseline. Because the risk of cardiovascular events increases with increasing blood pressure without any threshold,^1–3^ the continuous relationship between baseline ECG voltage and future increases in blood pressure provides important information in terms of preventing cardiovascular disease. Individual cardiovascular risk can thus be evaluated using this classical modality developed >100 years ago.

Our study was not designed to investigate the mechanism underlying the close relationship between LVH and the future incidence of hypertension in individuals without hypertension, and the observed relationship does not necessarily indicate a causal relationship. However, it is possible that during the development of hypertension, blood pressure increases gradually, with day-to-day or diurnal variation.^3,22,23^ Individuals with transient (but frequent) increases in blood pressure, especially when experiencing high levels of stress, are not generally identified as having hypertension by clinical examination, or may be identified as having “white coat” hypertension. However, such individuals could be on the way to developing hypertension. Indeed, individuals with white coat hypertension are at high risk of developing “true” hypertension.^24^ Our fundamental hypothesis is based on the speculation that even a mild and transient increase in blood pressure can result in an increased left ventricular mass, and that Sokolow–Lyon voltage and Cornell product criteria might be sensitive enough to identify such tiny changes in cardiac mass. In line with this speculation, the indices obtained from ECG recordings showed independent correlation with systolic blood pressure at baseline. BNP has also been reported as a sensitive marker of cardiac afterload,^25–27^ being closely correlated with central blood pressure, which reflects left ventricular afterload.^28^ We previously reported that plasma BNP levels could be used to predict the new onset of hypertension in the general Japanese population.^29^ However, the peptide level was not independently correlated with the incidence of hypertension because adjustment for baseline blood pressure abrogated the significant relationship between peptide level and incident hypertension.^29^ In contrast, significant correlations were observed between Sokolow–Lyon voltage and the incidence of hypertension and between Cornell product and the incidence of hypertension, even after adjustment for important factors including blood pressure at baseline. The difference in predictive value between LVH defined by ECG and BNP might, at least in part, be due to the short biological half-life of BNP (i.e., its fast elimination). Indeed, the half-life of BNP is <1 hour^25,30–32^; thus, it does not have sufficient predictive power to be used as a risk marker of future development of hypertension. In contrast, an increase in cardiac mass in the early stages of developing hypertension might be progressive but is unlikely to regress substantially. The fact that the risk of hypertension increases as these values obtained from ECG increase indicates an increased risk of hypertension with increasing chance of exposure to high blood pressure (afterload). There is another possible explanation for the present results. An increase in cardiac output might precede an increase in total peripheral resistance in the very early stage of developing hypertension,^33,34^ and thus, LVH detected by ECG could reflect the hyperkinetic state of the heart that produced the increased cardiac output.

The risk of hypertension is increased in individuals with prehypertension, family history of hypertension, obesity, impaired glucose tolerance, reduced glomerular filtration rate, and low-grade albuminuria.^35–37^ Furthermore, recent studies implicated some genetic factors in the development of hypertension.^38,39^ Our study adds new information to this field. Among the predictors of hypertension, indices obtained from ECG have a relatively large impact on incident hypertension as the presence of LVH defined by Sokolow–Lyon or Cornell product indicates an increase in the risk of hypertension of ∼50%. Moreover, ECG is not an expensive or complex clinical examination; thus, it can be performed routinely and is ideal for mass screening. Education in lifestyle modification for individuals at high risk of hypertension could contribute to effective primary prevention of hypertension.

Some caution should be taken in interpreting our data. The study subjects were participants in an annual physical check-up program, so blood pressure was measured only once a year; thus, false-positive or false-negative diagnoses of hypertension could not be completely excluded. The possibility that some participants developed secondary hypertension during the follow-up cannot be completely denied, although annual physical check-up did not reveal any suspicious findings of secondary hypertension. Close relationships of alcohol intake, physical activity, and dietary salt consumption with the incidence of hypertension have been demonstrated, but the present study did not have information about these factors. Finally, differences in baseline characteristics other than the ECG indices between participants with and without future development of hypertension may have affected future blood pressure, although these variables were adjusted for in the multivariate analyses.

In conclusion, Sokolow–Lyon voltage and Cornell product are novel predictors of future hypertension and increases in blood pressure in the general population. The risk of hypertension increases even below the threshold of Sokolow–Lyon voltage and Cornell product defined for LVH. These findings suggest that the amplitude of voltage in ECG has a close association with future blood pressure and is an important marker for managing blood pressure.
